# Gut microbiota-derived succinate: Friend or foe in human metabolic diseases?

**DOI:** 10.1007/s11154-019-09513-z

**Published:** 2019-10-25

**Authors:** Sonia Fernández-Veledo, Joan Vendrell

**Affiliations:** 1 Departament of Endocrinology and Nutrition and Research Unit, University Hospital of Tarragona Joan XXIII-Institut d’Investigació Sanitària Pere Virgili (IISPV), c/ Dr. Mallafré Guasch, 4, 43007 Tarragona, Spain; 2 CIBER de Diabetes y Enfermedades Metabólicas Asociadas (CIBERDEM)-Instituto de Salud Carlos III, Madrid, Spain; 3 Rovira i Virgili University, Tarragona, Spain

**Keywords:** Succinate, Microbiota, Metabolism

## Abstract

There is now a wealth of evidence showing that communication between microbiota and the host is critical to sustain the vital functions of the healthy host, and disruptions of this homeostatic coexistence are known to be associated with a range of diseases including obesity and type 2 diabetes. Microbiota-derived metabolites act both as nutrients and as messenger molecules and can signal to distant organs in the body to shape host pathophysiology. In this review, we provide a new perspective on succinate as a gut microbiota-derived metabolite with a key role governing intestinal homeostasis and energy metabolism. Thus, succinate is not merely a major intermediary of the TCA traditionally considered as an extracellular danger signal in the host, but also a by-product of some bacteria and a primary cross-feeding metabolite between gut resident microbes. In addition to maintain a healthy microbiome, specific functions of microbiota-derived succinate in peripheral tissues regulating host nutrient metabolism should not be rule out. Indeed, recent research point to some probiotic interventions directed to modulate succinate levels in the intestinal lumen, as a new microbiota-based therapies to treat obesity and related co-morbidities. While further research is essential, a large body of evidence point to succinate as a new strategic mediator in the microbiota-host cross-talk, which might provide the basis for new therapeutically approaches in a near future.

## Introduction

Gut microbiota – the complex ecosystem of trillions of microorganisms that inhabit our gastrointestinal tract – has a profound role in shaping the physiology of the healthy host, especially gut maturation, nutrient acquisition and energy metabolism, and the immune system [1, 2]. It is well known that compositional and metabolic changes to the gut microbiota – termed *dysbiosis* – are associated with diverse pathological processes. Indeed, increasing evidence points to gut microbiota dysbiosis as a determining factor in the etiology of several diseases, both intestinal such as inflammatory bowel disease (IBD), and extra-intestinal, such as obesity, type 2 diabetes, non-alcoholic fatty liver disease and cancer [3–5]. However, whether there is a direct causal relationship between microbiota dysbiosis and disease, or whether the former is a consequence of the latter, remains uncertain in humans [6–10]. In this context, products of bacterial metabolism have been linked both to intestinal health and disease.

**Fig. 1:**
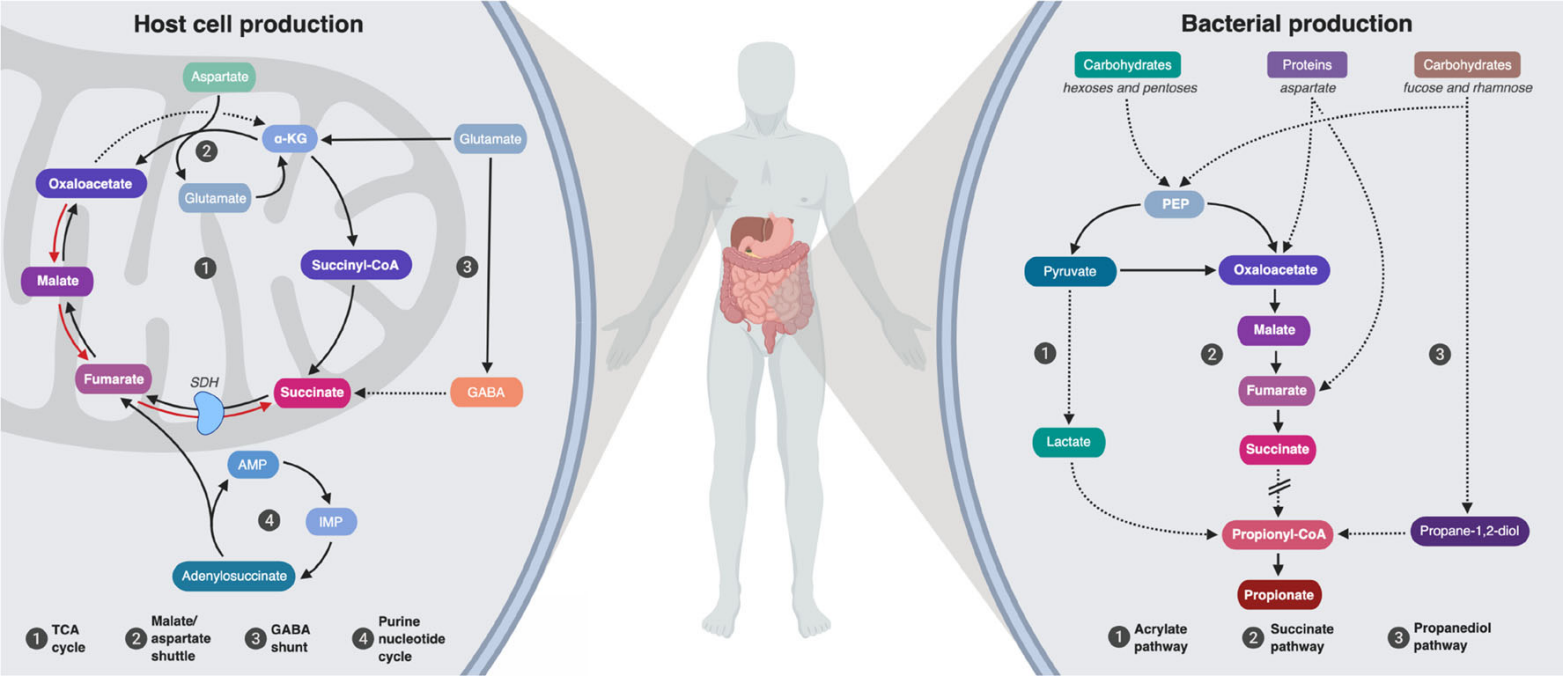
Succinate synthesis by host and gut microbiota. (**left**) Succinate is a tricarboxylic acid (TCA) cycle intermediary metabolite produced in the mitochondria of host cells. Succinate occupies a pivotal position in host metabolism as the only direct link between the TCA cycle and the mitochondrial respiratory chain through reversible succinate dehydrogenase (respiratory chain complex II) activity. Nonetheless, when cells rely on anaerobic glycolysis, in hypoxic conditions, or upon activation of certain innate immune cells, mitochondrial levels of succinate might increase by alternative metabolic pathways such as reverse succinate dehydrogenase activity, glutamine-dependent anaplerosis and the gamma-aminobutyric acid (GABA) shunt. Succinate is then released into the cytosol and the extracellular space, where it can act as a signaling metabolite. (**right**) Succinate is also a catabolic metabolite of microbial carbohydrate fermentation. The succinate pathway is the most prevalent biochemical pathway of propionate production by primary fermenters. Succinate and propionate can also be formed as metabolites from amino acid fermentation. Succinate is not only a common by-product of some bacteria, but it also a key cross-feeding metabolite since it can be consumed by secondary fermenters (see Table 1). TCA, tricarboxylic acid. (1) Acrylate pathway, (2) Succinate pathway, (3) Propanediol pathway. Solid line: direct reaction; dotted line: multiple reactions

In this review, we focus on the tricarboxylic acid (TCA) cycle metabolite succinate, which is quickly becoming a poster child for microbiota-derived metabolites with important roles in gut homeostasis, pathogen susceptibility and inflammatory-related diseases such as IBD and obesity. Notably, succinate has the distinction of being produced by both the microbiota and the host (Fig. 1), placing it in the unique position of being at the interface of host-gut microbiota metabolic interactions. Rather than an exhaustive summary of the literature, our goal in this review is to provide some key concepts and highlight existing questions in relation to succinate as a friend or foe in microbiota-related health and disease. We apologize in advance to our colleagues whose work has been omitted unintentionally and due to space constraints.

## Succinate, a metabolite with pleiotropic functions

In host cells, succinate is best known as an intermediate of the TCA or Krebs cycle and is traditionally considered as a fuel substrate for mitochondrial oxidative phosphorylation (Fig. 1). More recently, succinate has received growing attention as a signaling molecule both in the cytosol and extracellularly. In an ever-expanding list of processes, studies have demonstrated that the accumulation of succinate in the cytosol is directly related to protein post-translational modifications by succinylation, stabilization of the hypoxia-inducible factor family of transcription factors and activation of pro-inflammatory programs, epigenetic regulation and reactive oxygen species production [11–13]. There is also the general assumption that succinate acts as a pro-inflammatory stimulus [12, 14, 15] to regulate local stress, tissue damage and immune response [11, 13, 16, 17]. On top of this, there is some evidence supporting the beneficial effects of intracellular succinate as a modulator of intestinal gluconeogenesis [18–20] and thermogenesis [21].

At the extracellular level, succinate is sensed by its cognate receptor SUCNR1 (GPR91), a G protein-coupled receptor (GPCR) expressed on the plasma membrane of a broad range of cells [22–24]. As with other GPCRs, SUCNR1 transmits signals *via* multiple pathways in a cell-specific manner [25]. Our knowledge about the signaling and function of this receptor is, nevertheless, limited. As mentioned above, succinate has long been perceived as a stress-induced signaling mediator that boosts pro-inflammatory responses for optimal immune activation [14, 15, 26]. Conversely, anti-inflammatory functions have also recently been ascribed to SUCNR1 through its activation in neural stem cells [27]. Similarly, a recent study from our laboratory has uncovered a hitherto unrecognized mechanism whereby SUCNR1 signaling in macrophages is key for the active resolution of acute inflammation in the context of obesity – a physiological circuit broken in human obesity [28]. Interestingly, obesity is associated with higher circulating levels of succinate [29] but impaired SUCNR1-signaling, which we have termed a succinate-resistant state [28]. Remarkably, succinate-SUCNR1 signaling has been described as a major driver of helminth-triggered type 2 immunity in the intestine [30], although the source of succinate (microbiota, diet or dying epithelial cells) remains to be determined. While the available evidence supporting succinate as a harmful or a beneficial signal is inconclusive, it would seem fairly evident that the succinate/SUCNR1 axis might serve as a link between metabolic stress and immune function [28, 31, 32].

The role of succinate in the metabolic regulation of immune cells has been extensively reviewed by others [13, 33–35]; yet, despite significant progress in recent years, further research is necessary to build a complete picture of both intracellular and extracellular functions of succinate, not only in immune cells but also in other tissues and organs beyond the immune system. This is important because, as a GPCR, SUCNR1 is a highly druggable target accessible with available small molecules [36–39]. Accordingly, the physiological and pathological functions of extracellular succinate deserve further investigation to evaluate the potential of its receptor as a pharmacological target. Indeed, a bulk of evidences point to SUCNR1 as a masterpiece in the etiology of some disturbances associated with obesity and T2D. Extracellular succinate induces inhibition of lypolisis in adipose tissue [31, 40] and SUCNR1-signalling seems to be a major regulator of blood pressure in T2D by a mechanism dependent on activation of the renin-angiotensin system (RAS) [17]. Moreover, SUCNR1 signaling plays a key role in diabetic retinopathy through the induction of retinal neovascularization by VEGF [41] and has pathological implications in hypertrophic cardiomyopathy [42], steatohepatitis and liver fibrosis [43, 44]. For a comprehensive review on SUCNR1-succinate signaling, see [22, 23].

Circulating levels of succinate are elevated in several physiological conditions such as endurance exercise [45], and also in some metabolic- and inflammatory-related diseases, including ischemic heart disease [42], hypertension [46], type 2 diabetes [29, 32] and obesity [29]. Nonetheless, the origin of circulating succinate remains vague. While it is plausible that tissue damage contributes to the succinate found in circulation in a pathological context, a microbial origin of succinate should not be ruled out. Along this line, we recently provided the first demonstration of a close relationship between circulating succinate and gut microbiota in human obese subjects [29]. The following sections are intended to address succinate as a microbiota-produced metabolite, which might play a key role in both intestinal and extra-intestinal diseases associated with microbiota dysbiosis.

## Succinate, a common by-product of microbiota

The gut microbiota metabolizes different dietary and host-derived nutrients and produces end products that can be absorbed by the host, for example, short-chain fatty acids (SCFAs) and organic anions (lactate and succinate). Specifically, dietary non-digestible carbohydrates are the main source in the production of the SCFAs acetate, propionate and butyrate, which are considered the most common end products of microbial fermentation [47, 48]. Although SCFAs have diverse effects on host physiology, they essentially confer a range of health-promoting functions by acting as key energy substrates for colonocytes, enterocytes and hepatocytes, while at the same time acting as signaling molecules recognized by specific GPCRs targeting primarily enteroendocrine and immune cells in the lamina propia of gut mucosa [49–51].

Succinate is a metabolic end-product of some bacteria, but it has been classically overlooked and has only been considered as a key intermediate in microbial propionate synthesis (Fig. 1). Propionate is synthesized *via* two independent microbial pathways. The majority of pentose and hexose carbohydrates are fermented through the succinate pathway, especially in Bacteroidetes and in the Negativicutes class of Firmicutes [52], whereas the deoxy sugars are processed *via* the propanediol pathway [53]. However, succinate is not only a precursor of propionate – commonly produced by primary fermenters such as Bacteroides – but it is also consumed by secondary fermenters. Thus, an accumulation of succinate in cultures of some Bacteroides spp. has been described under specific growth conditions such as high concentrations of CO_2_ [54], and also in cultures of *Prevotella ruminocola* grown in the absence of vitamin B12 [55]. Remarkably, an increase in the levels of cecal succinate was described in conventional mice colonized with the succinate producer *Prevotella copri* [18]. Similarly, some Ruminococcaceae, such as *Ruminococcus flavefaciens*, have been described as succinate-producing bacteria [56]. Conversely, some human colonic bacteria belonging to the Negativicutes class of Firmicutes, such as *Phascolarctobacterium succinatutens* [57], possess the capacity to convert succinate to propionate [52, 58]. There is no standard classification for succinate producers and consumers, although succinate has been described as an excreted/consumed product for some bacterial species (see Table 1). It should be noted, however, that most of these studies are based on cells grown in culture where cross-feeding relationships (for example, the exchange of nutrients) are absent. In relation to gut microbiota, it is clearly important to appreciate how the different ecological niches of the community interact in terms of metabolism, and how this could be used to better understand the role of microbial metabolites such as succinate in physiology and in dysbiosis-related diseases.

**Table 1:** Bacterial species referred to as succinate-producers or succinate-consumers

SPECIES	FAMILY	PHYLUM	References
Succinate-producers			
*Propionibacterium acidipropionici*	Propionibacteriaceae	Actinobacteria	[87]
*Propionibacterium shermanii*	Propionibacteriaceae	Actinobacteria	[88]
*Bacteroides fragilis*	Bacteroidaceae	Bacteroidetes	[88, 89]
*Alistipes indistinctus*	Rikenellaceae	Bacteroidetes	[90]
*Bacteroides vulgatus*	Bacteroidaceae	Bacteroidetes	[58, 73]
*Paraprevotella clara*	Prevotellaceae	Bacteroidetes	[91]
*Paraprevotella xylaniphila*	Prevotellaceae	Bacteroidetes	[57, 91]
*Parabacteroides distasonis*	Tannerellaceae	Bacteroidetes	[20]
*Blautia wexlerae*	Lachnospiraceae	Firmicutes	[58]
*Faecalibacterium prausnitzii*	Ruminococcaceae	Firmicutes	[92]
*Ruminococcus albus*	Ruminococcaceae	Firmicutes	[88]
*Citrobacter freundii*	Enterobacteriaceae	Proteobacteria	[93]
*Succinivibrio dextrinosolvens*	Succinivibrionaceae	Proteobacteria	[94]
*Akkermansia muciniphila*	Verrucomicrobiaceae	Verrucomicrobia	[84]
Succinate-consumers			
*Bacteroides thetaiotaomicron*	Bacteroidaceae	Bacteroidetes	[19, 78]
*Phascolarctobacterium faecium*	Acidaminococcaceae	Firmicutes	[95, 96]
*Phascolarctobacterium succinatutens*	Acidaminococcaceae	Firmicutes	[57]
*Ruminococcus bromii*	Ruminococcaceae	Firmicutes	[97]
*Dialister propionicifaciens*	Veillonellaceae	Firmicutes	[98]
*Dialister succinatiphilus*	Veillonellaceae	Firmicutes	[99]
*Veillonella parvula*	Veillonellaceae	Firmicutes	[100]

## Microbiota-derived succinate in health and disease

In a generally healthy status, colonic and cecal concentrations of SCFAs range from 1 to 3 mM, whereas in circulation the concentrations of these metabolites are in the micromolar range. At the systemic level, acetate is the most abundant SCFA (5–200 μM), followed by propionate and butyrate (≤12–13 μM) [5, 34]. By comparison, succinate is detected at a relatively low concentration in the gut lumen, likely related to its conversion to propionate by cross-feeding between different gut bacteria [59]. Studies comparing germ-free mice with control mice have shown that fecal succinate levels are almost undetectable in the former, which points to gut microbiota as the predominant source of luminal succinate [60–62].

In the context of disease, several studies have revealed a clear association between gut microbiota disturbances, linked for example to antibiotic-induced dysbiosis [63, 64], motility disturbances [65] and specially IBD [34, 66, 67], and succinate accumulation in the gut lumen. More specifically, there is a wealth of evidence, both in mice and humans, demonstrating that IBD causes an increase in fecal succinate, which has been related to disease activity [68–71]. While the contribution of intestinal damage *versus* gut microbiota dysbiosis to this increase in succinate is not clear, a metagenomic study of the gut microbiome of patients with IBD reported a significant decrease in the levels of specific succinate-consuming bacterial strains [72]. By contrast, an increase of succinate-producing Bacteroides has been described in a chemically-induced model of colitis in mice [71]. Remarkably, colonization of germ-free mice with succinate-producing bacteria from IBD patients worsens intestinal inflammation in a mouse model of dextran sulfate sodium-induced colitis, which is associated with higher levels of fecal succinate [73]. Thus, the available evidence suggests a link between dysbiosis, succinate accumulation in gut, and inflammation. However, whether this scenario is directly related to disease outcomes is less clear.

By analogy to SCFAs, it is not unreasonable to expect that levels of circulating succinate might depend on diet, microbiota composition and also splenic extraction ratio. This might be particularly relevant in pathological conditions associated with alterations in intestinal barrier function (leaky gut). Indeed, an increase in both serum and intestinal succinate levels has been reported in patients with Crohn’s disease when compared with healthy subjects [66]. Similarly, higher succinate levels have been reported in breast milk from mothers with IBD as compared with healthy peers [74]. Interestingly, we recently reported a strong association between microbial gut flora and circulating succinate in humans [29]. Using a multi-cohort analysis of the intestinal metagenome based on DNA extracted from fecal samples, we identified a specific intestinal bacterial signature – the ratio of succinate producers (*Prevotellaceae + Veillonellaceae*) *versus* consumers (*Odoribacteraceae* + *Clostridaceae*) – as a main determinant of plasma succinate, which was higher in obese patients than in controls. We also established that modification of the gut microbiota by dietary weight loss intervention, but also microbiota spontaneous evolution independent of body weight, drives changes to the levels of circulating succinate, which are closely linked to a specific molecular entity and metabolic function of microbiota related to succinate metabolism [29]. Although we could not demonstrate a causal relationship between succinate levels and disease (in this case, obesity), all evidence points to succinate as a new player in the pathophysiology of obesity-related metabolic disturbances.

**Fig. 2:**
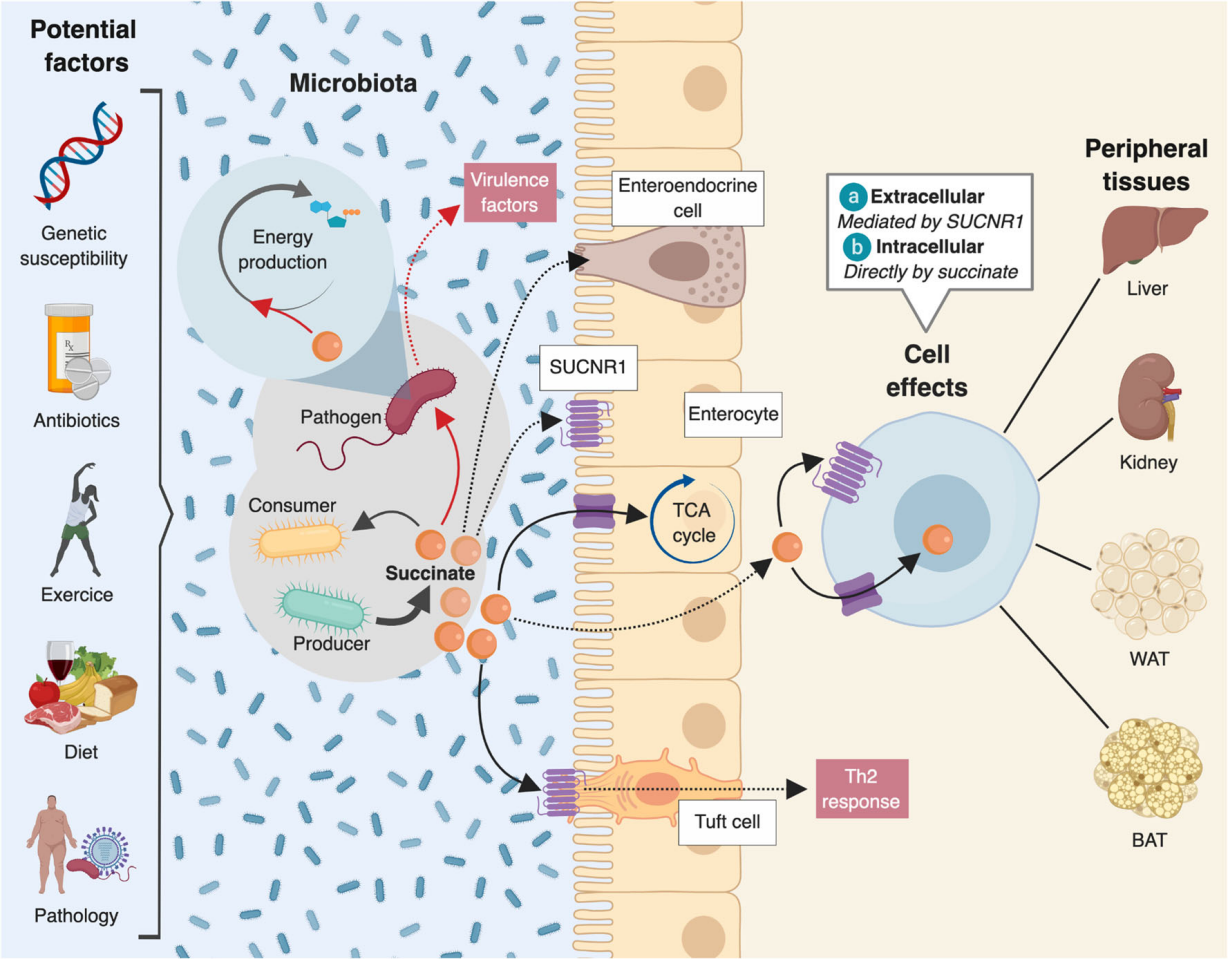
Succinate as a potential intermediate in host-microbiota interactions. The intestinal efflux by specific gut microbiota, which can be affected by several endogenous and exogenous factors, might be a relevant source of circulating succinate. Similar to other metabolites, succinate might act as a fuel or signaling metabolite in host peripheral tissues regulating energy metabolism. Succinate has been reported to trigger intestinal gluconeogenesis, and type 2 immunity *via* SUCNR1 activation in Tuft cells. Whether gut microbiota-derived succinate might have other metabolic and immune effects in peripheral tissues cannot be excluded. Indeed, succinate has recently emerged as metabolite controlling activation of brown adipose tissue and some metabolic functions have been assigned to SUCNR1-signalling in other tissues (e.g negative regulator of lipolysis in adipose tissue, activator of renal renin-angiotensin system). In addition, the role of succinate in intestinal lumen on the microbial ecosystem might be of special relevance. Thus, succinate has emerged as a cross-feeding metabolite with a key role in the reconstitution of the gut bacterial ecosystem but also in commensal-pathogen interactions

It is known that microbiota dysbiosis can provide a competitive advantage to enteric pathogens. Moreover, the metabolic functions of each specific bacterial strain are directly related to the community structure. In this context, CO_2_ levels seem to be a decisive factor in the selection of succinate or propionate as a microbial by-product [75]. That said, few studies have specifically examined the effects of succinate on maintenance and resilience of gut microbiota, with most ascribing a detrimental role to succinate (Fig. 2). Earlier published works described succinate as a virulence factor that might exacerbate enteric infections [76, 77]. As mentioned above, conventional mice have low levels of succinate in the gut lumen. However, antibiotic treatment or chemically-induced intestinal motility disturbances leads to a transient increase in its concentration, which could be exploited by *Clostridium difficile* (a primary cause of antibiotic-associated diarrhea) to efficiently proliferate [65]. Likewise, succinate-rich environments are sensed by enterohemorrhagic pathogens such as *Escherichia coli* and *Citrobacter rodentium* to activate virulence factors [78]. In addition, the mucosal inflammatory response triggered by *Salmonella typhimurium* induces a metabolic adaptation in the pathogen itself to use microbiota-derived succinate as a nutrient for colonization of the intestinal tract [79].

Overall, it is clear that succinate plays a crucial role in commensal-pathogen interactions. Nonetheless, as recently described by Kim and collaborators, succinate might also have beneficial effects on bacterial ecosystems in neonatal states. In the context of the neonatal gut, Bacteroides-derived succinate might favor the colonization by strict anaerobes such as Clostridia spp., which prevents the growth of diarrhea-causing pathogens such as *S. typhimurium* [80]. Clearly, further research is needed to more fully understand the role of succinate in the gut ecosystem. It might be conceivable that succinate acts as a primary cross-feeding metabolite essential for the maintenance of a healthy resident gut microbiota. In this sense, the increase in succinate in some pathological conditions would be reflective of dysbiosis, but would also be exploited by some pathogens.

## Could probiotic interventions directed to modulate gut-derived succinate be used to treat obesity-related disorders?

While most published data link a succinate-enriched gut environment to pathological states associated with dysbiosis, several recent studies have described the metabolic benefits of specific commensal bacteria *via* succinate production, particularly in obesity-related metabolic disturbances. For instance, Kovatcheva-Datchary and colleagues found that the gut microbiota of those healthy subjects showing an improvement in glucose metabolism following consumption of barley kernel-based bread was enriched for *P. copri* – a well-established succinate producer [19]. Moreover, microbiota from these subjects improved glucose metabolism in germ-free mice when compared with similar mice transplanted with the microbiota of non-responders to barley kernel-based bread consumption. Accordingly, colonization of standard diet-fed mice with *P. copri* increased cecal succinate and improved glucose tolerance [19], a metabolic effect not detected with a propionate producer such as *Bacteroides thetaiotaomicron.* However, the authors concluded that the high levels of succinate observed in *P. copri-*treated mice were not sufficient to explain the metabolic beneficial effects of this bacterial strain since co-colonization with a *B. thetaiotaomicron* mutant unable to convert succinate to propionate failed to improve glucose response [19].

A subsequent study from the same group reported that colonization with *P. copri* inhibits hepatic glucose production in mice [18]. Moreover, succinate has been revealed as an important microbial product for the beneficial metabolic effects of dietary fiber consumption, which increases Prevotella-produced succinate [81–83]. Interestingly, the abundance of *Akkermansia muciniphila*, which produces succinate as one of the major metabolites from mucin degradation [84]*,* has been systemically found to be inversely correlated with obesity-related metabolic disturbances. Indeed, some prebiotic interventions that improve metabolic disorders associated with obesity, antidiabetic drugs such as metformin, and bariatric surgery, are associated with an increase in the abundance of *A. muciniphila* [85]. Colonization with other succinate producers such as *Parabacteroides distasonis* has also proven effective for improving metabolic dysfunctions associated with obesity [20]. Interestingly, the abundance of this commensal bacterium has been negatively correlated with IBD, which has been widely demonstrated to be linked to elevations in cecal succinate levels [34, 66, 67]. Nonetheless, secondary bile acids have also been identified as a potential mechanism involved in the metabolic beneficial effects of *P. distasonis* [20].

The potential for probiotics to be used in the management of metabolic disorders including obesity and type 2 diabetes is becoming an important research topic. Indeed, the first studies exploring this support succinate as a potential target microbial-metabolite [18, 19]. Nevertheless, given that these diseases are generally associated with higher succinate levels, further investigation is needed to clarify the mechanisms of succinate biology. Whether this perspective applies to other dysbiosis-related diseases should also be considered in future research.

## Conclusions and future perspectives

Recent research has shed new light on the TCA cycle intermediate succinate, which is now recognized both as a fuel and as a signaling metabolite with unexpected pleiotropic functions, such as a positive regulator of intestinal gluconeogenesis [18] and thermogenesis [21], as well as a key mediator in the resolution of inflammation associated with obesity [28]. Intriguingly, unlike other intermediary metabolites, succinate holds the unique distinction of being at the interface of host-gut microbiota metabolic interactions. Accordingly, succinate has emerged as a gut microbial-derived metabolite associated with dysbiosis-related diseases such as obesity and IBD. Moreover, its contribution to cross-feeding interactions might classify it as a primary metabolite essential for the stability and resilience of gut microbiota. While tremendous strides are currently being made in our understanding of succinate function, much work remains to be done both in mice and humans before claiming succinate as a microbial-derived metabolite with potential health-promoting effects. Moreover, and similar to other microbiota-derived metabolites such as SCFAs [51, 86], succinate might reach the circulation and act as a signaling metabolite in peripheral tissues (Fig. 2), where it may have yet unidentified functions. Finally, the use of probiotics to shape the gut microbiota has the potential to herald a new era in the management of metabolic diseases linked to microbiota dysbiosis, such as obesity, and succinate may serve as a promising metabolite target for this approach.
